# Prognostic value and therapeutic potential of the long noncoding RNA TP73-AS1 in cancers: A systematic review and meta-analysis

**DOI:** 10.1038/s41598-020-65726-2

**Published:** 2020-06-03

**Authors:** Yuan Zhong, Meng Zhao, Yang Yu, Quanpeng Li, Fei Wang, Peiyao Wu, Wen Zhang, Lin Miao

**Affiliations:** 1 Medical Center for Digestive Diseases, the Second Affiliated Hospital of Nanjing Medical University, Nanjing, 210011 China; 2 Department of Cardiovascular Surgery, the First Affiliated Hospital of Nanjing Medical University, Nanjing, 210011 China; 3 Nantong Tumor Hospital, Nantong, 226300 China

**Keywords:** Cancer, Prognostic markers

## Abstract

Studies published in recent years have demonstrated that abnormal long noncoding RNA (lncRNA) antisense RNA to TP73 gene (TP73-AS1) expression is markedly associated with tumorigenesis, cancer progression and the prognosis of cancer patients. We aimed to explore the prognostic value of TP73-AS1 in multiple cancers. We comprehensively searched PubMed, Embase, Web of Science and the Cochrane Library (up to February 21, 2019). Hazard ratios (HRs), odds ratios (ORs) and the corresponding 95% confidence intervals (95% CIs) were calculated to estimate the association of TP73-AS1 with survival and clinicopathological features. The potential targets and pathways of TP73-AS1 in multiple cancers were summarized. Nineteen studies that involved thirteen types of cancers and 1329 cancer patients were identified as eligible for this meta-analysis. The results showed that high TP73-AS1 expression was significantly correlated with shorter overall survival (OS) (HR = 1.962, 95% CI 1.630-2.362) and disease-free survival (DFS) (HR = 2.050, 95% CI 1.293-3.249). The summary HRs of OS were 2.101 (95% CI 1.516-2.911) for gastric cancer (GC) and 1.920 (95% CI 1.253-2.942) for osteosarcoma. Subgroup analysis of OS demonstrated that the differential expression of TP73-AS1 in cancer tissues was a potential source of heterogeneity. Furthermore, increased TP73-AS1 expression was markedly associated with larger tumor size (OR = 2.759, 95% CI 1.759-4.330), advanced histological grade (OR = 2.394, 95% CI 1.231-4.656), lymph node metastasis (OR = 2.687, 95% CI 1.211-5.962), distant metastasis (OR = 4.145, 95% CI 2.252-7.629) and advanced TNM stage (OR = 2.633, 95% CI 1.507-4.601). The results of Egger’s test and sensitivity analysis verified the robustness of the original results. High TP73-AS1 expression can predict poor survival and poor clinicopathological features in cancer patients and TP73-AS1 might be a potential biomarker and therapeutic target.

## Introduction

Cancer is an essential public health problem and a major cause of death worldwide. In 2019, Approximately 1,762,450 cancer cases will be diagnosed and 606,880 cancer patients will die in America as estimated by the American Cancer Society^1^. Although great advances in diagnoses and treatments have improved survival, the prognosis of patients with advanced cancers remains poor^2^. The exploration of novel therapeutic targets and prognostic biomarkers is a top priority. LncRNAs, comprising more than 200 nucleotides, are engaged in many biological processes, such as cell proliferation, differentiation, the DNA damage response and chromosomal imprinting^3,4^. A large number of studies have suggested that lncRNAs are tightly linked with oncogenicity and cancer progression^5–7^.

An antisense RNA to the TP73 gene (TP73-AS1), also named *KIAA0495*/PDAM, is located in the 1p36 chromosome region that is frequently deleted in tumors and comprises tumor suppressor genes^8^. TP73-AS1 functions as a tumor suppressor by sponging miR-941 at nine high-affinity binding sites^9^. TP73-AS1 can suppress the progression of bladder cancer by epithelial-mesenchymal transition (EMT), and low TP73-AS1 expression predicts a shorter survival of bladder cancer patients^10^. However, TP73-AS1 also acts as a tumor promoter. For example, TP73-AS1 can promote hepatocellular carcinoma (HCC) cell proliferation via miR-200a-dependent HMGB1/RAGE regulation, and its high expression level is significantly associated with a poorer prognosis of HCC patients^11^. Knockdown of TP73-AS1 suppresses the proliferation and invasion of osteosarcoma cells by sponging miR-142^12^. TP73-AS1, which is upregulated in cholangiocarcinoma (CCA) tissues, predicts adverse phenotypes for CCA, and silencing TP73-AS1 attenuates CCA cell proliferation, migration and invasion^13^.

Therefore, we performed this meta-analysis based on studies focusing on the association of TP73-AS1 with survival and clinicopathological features. We aimed to explore the potential of TP73-AS1 as a novel biomarker and therapeutic target.

## Materials and methods

### Search strategy

Two authors (Yuan Zhong and Yang Yu) independently screened PubMed, Web of Science, the Cochrane Library and Embase up to February 21,2019. The search terms were used as follows: (“TP73-AS1” OR “P73 antisense RNA 1 T” OR “antisense RNA to TP73 gene” OR “KIAA0495” OR “PDAM”) AND (“cancer” OR “tumor” OR “carcinoma” OR “neoplasm”).

### Selection criteria

Inclusion criteria were as follows: (1) TP73-AS1 expression was detected in cancer tissues; (2) Patients were divided into two groups based on TP73-AS1 expression levels; (3) The association between TP73-AS1 expression and the prognosis of patients with cancer was investigated; and (4) Sufficient data were extracted to compute the HR and 95% CI of survival or the OR and 95% CI of clinicopathological parameters. Exclusion criteria were as follows: (1) Nonhuman studies, reviews, editorials, expert opinions, letters, and case reports; (2) Studies lacking key data; and (3) Duplicate publications.

### Data extraction and quality assessment

Two authors extracted the following requisite data in each qualified article: first author’s name, publication year, country, cancer type, sample type, sample size, detection method, cut-off value, the number of patients in the high and low TP73-AS1 expression groups, survival analysis method, the HR and 95% CI of TP73-AS1 for survival and data for age, gender, TNM stage, tumor size, lymph node metastasis, etc. If the HR was not accessible from the original text, we utilized Engauge Digitizer v.10.11 software to extract data from the Kaplan-Meier survival curve and calculated the HR with assistance from Tierney’s spreadsheet^14^. The HR with the 95% CI from multivariate analysis was preferred over that from the univariate analysis because the multivariate analysis considered the effects of other factors. Two authors independently assessed the quality of each eligible study under the guidance of the Newcastle-Ottawa Quality Assessment Scale (NOS)^15^.

### Statistical analysis

Pooled HRs with 95% CIs were calculated to investigate the value of TP73-AS1 on the survival of patients with cancer. Pooled ORs with 95% CIs were used to evaluate the association between TP73-AS1 expression and clinicopathological features. The heterogeneity of the pooled results was explored by means of the Q test and I^2^ statistics. The fixed pooling model was selected when I^2^ < 50% and p > 0.10. Otherwise, the random pooling model was used. Subgroup analysis was further conducted to explore the potential sources of heterogeneity. Sensitivity analysis was performed to test whether each single study could influence the stability of the results. Egger’s test was utilized to assess a publication bias. A p value less than 0.05 was considered statistically significant.

## Results

### Characteristics of the included studies

**Figure 1:**
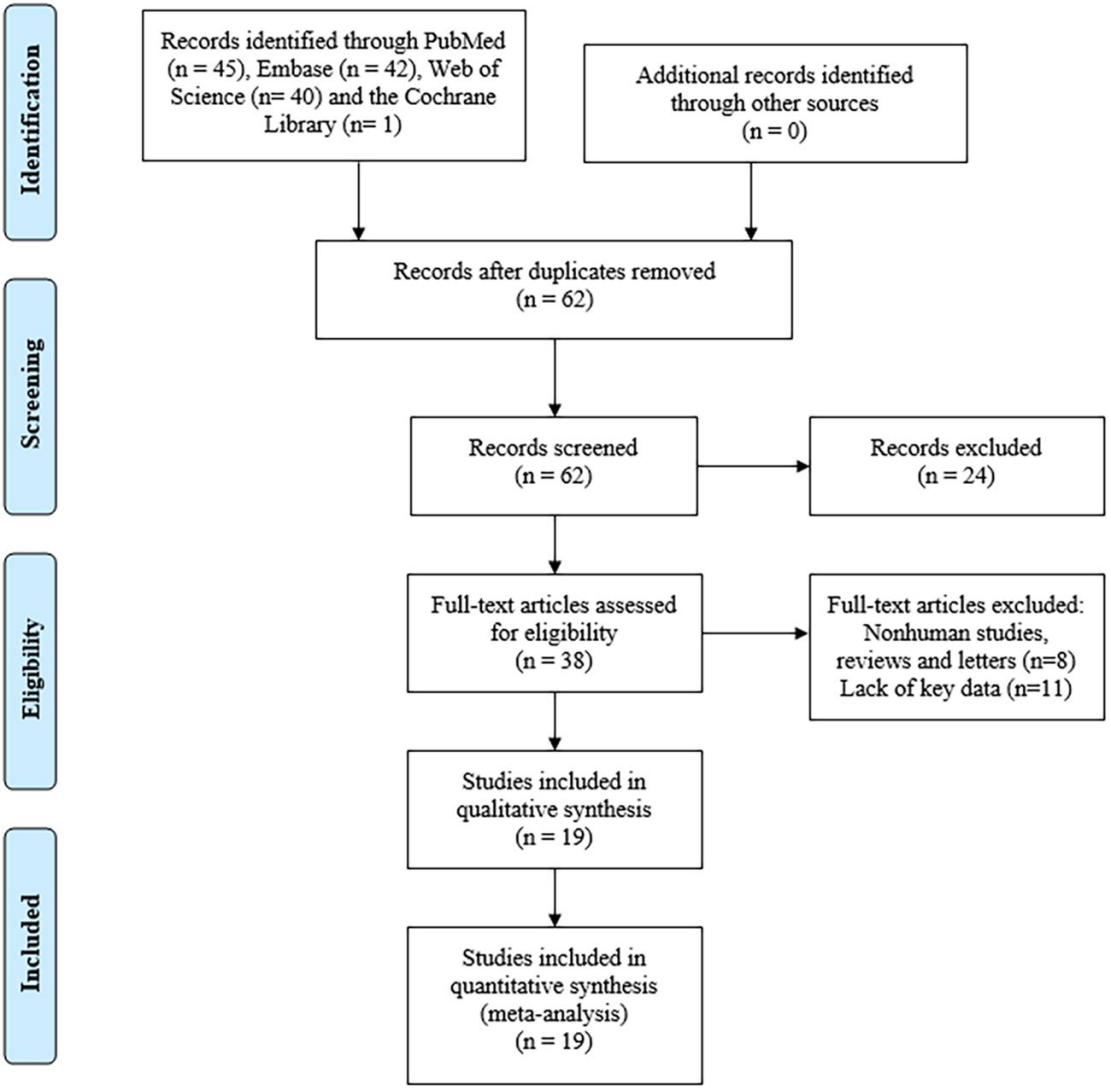
Flow diagram of this meta-analysis.

A total of 19 articles were eligible for this meta-analysis according to the selection criteria^9–13,16–27^. In total, 1329 patients with 13 types of cancer, including GC^9,16–18^, breast cancer^13,19^, ovarian cancer^20,21^, osteosarcoma^12,22^, brain glioma^23^, hepatocellular carcinoma (HCC)^11^, cholangiocarcinoma (CCA)^13^, colorectal cancer (CRC)^27^, pancreatic cancer^24^, lung adenocarcinoma (LAD)^25^, non-small cell lung cancer (NSCLC)^19^, bladder cancer^10^ and clear cell renal cell carcinoma (ccRCC)^26^, were analyzed. Twelve articles chose overall survival (OS) as the only survival outcome^9,11–13,17–19,21–25^, 2 articles used both OS and disease-free survival (DFS)^16,26^, and one article used both OS and progression-free survival (PFS)^10^. All studies were performed in China. TP73-AS1 expression levels were measured by quantitative real-time PCR (qRT-PCR). Patients were classified into high or low expression groups according to the cut-off value. More details are shown in Fig. 1 and Table 1.

**Table 1:** Characteristics of the included studies.

Cancer	First author	Year	Country	Sample type	Sample size(n)	Detection method	Cut-off value	PT	Survival analysis	Multivariate analysis	Hazard ratios	NOS score
GC	Wei Zhang^9^	2018	China	Tissue	76	qRT-PCR	NA	no	OS	No	K-M curve	7
GC	Yufeng Wang^16^	2018	China	Tissue	64	qRT-PCR	NA	NA	OS, DFS	No	K-M curve	6
GC	Jianjun Peng^17^	2018	China	Tissue	58	qRT-PCR	mean	no	OS	Yes	K-M curve	6
GC	Zhi Ding^18^	2018	China	Tissue	72	qRT-PCR	median	no	OS	no	K-M curve	6
Breast cancer	Qiongyan Zou^19^	2017	China	Tissue	86	qRT-PCR	median	NA	NA	No	NA	8
Breast cancer	Jia Yao^13^	2017	China	Tissue	36	qRT-PCR	2-fold	NA	OS	Yes	Text	7
Ovarian cancer	Xiaoqian Wang^20^	2018	China	Tissue	60	qRT-PCR	median	NA	NA	No	NA	8
Ovarian cancer	Xiuyun Li^34^	2018	China	Tissue	62	qRT-PCR	mean	no	OS	Yes	K-M curve	6
Osteosarcoma	Xi Chen^35^	2018	China	Tissue	132	qRT-PCR	NA	no	OS	Yes	Text	7
Osteosarcoma	Guangling Yang^12^	2018	China	Tissue	46	qRT-PCR	mean	no	OS	No	K-M curve	8
Brain glioma	Rong Zhang^23^	2017	China	Tissue	47	qRT-PCR	median	NA	OS	Yes	Text	7
HCC	Shaling Li^11^	2017	China	Tissue	84	qRT-PCR	median	NA	OS	no	K-M curve	8
Pancreatic cancer	Xianping Cui^24^	2019	China	Tissue	77	qRT-PCR	NA	NA	OS	No	K-M curve	7
LAD	Chunfeng Liu^25^	2019	China	Tissue	80	qRT-PCR	median	no	OS	Yes	K-M curve	7
NSCLC	Lin Zhang^19^	2018	China	Tissue	45	qRT-PCR	median	NA	OS	No	K-M curve	7
Bladder cancer	Zhiyong Tuo^10^	2018	China	Tissue	128	qRT-PCR	median	no	OS, PFS	No	K-M curve	7
ccRCC	Guanghua Liu^26^	2018	China	Tissue	40	qRT-PCR	median	no	OS, DFS	No	K-M curve	8
CCA	Yue Yao^13^	2018	China	Tissue	75	qRT-PCR	mean	no	NA	No	NA	8
CRC	Zeming Jia^27^	2019	China	Tissue	61	qRT-PCR	NA	no	NA	No	NA	7

Abbreviations: GC, gastric cancer; HCC, hepatocellular carcinoma; LAD, lung adenocarcinoma; NSCLC, non-small cell lung cancer; ccRCC, clear cell renal cell carcinoma; CCA, cholangiocarcinoma; CRC, colorectal cancer; PT, preoperative treatment; NA, Not available.

### Association between TP73-AS1 and survival

**Figure 2:**
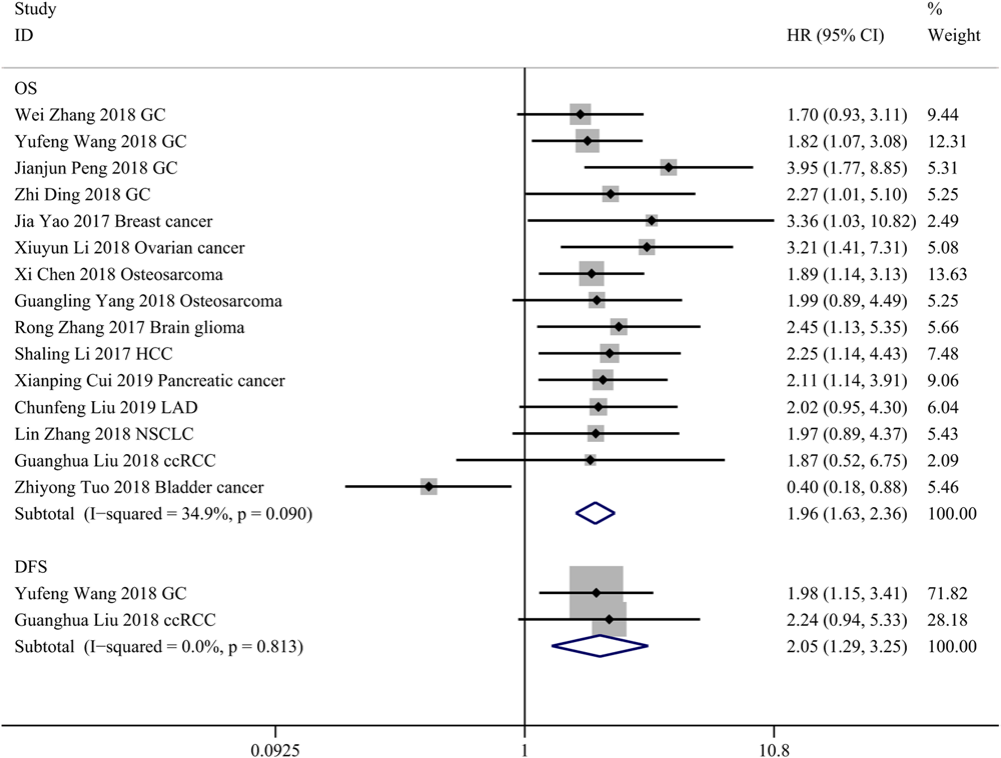
Forest plots for association of TP73-AS1 expression with overall survival (OS) and disease-free survival (DFS). Abbreviations: GC, gastric cancer; HCC, hepatocellular carcinoma; LAD, lung adenocarcinoma; NSCLC, non-small cell lung cancer; ccRCC, clear cell renal cell carcinoma.

TP73-AS1 was relatively upregulated in most cancer tissues compared to paired normal or non-cancerous tissues except for bladder cancer specimens. As shown in Fig. 2, high TP73-AS1 expression was significantly associated with poor OS (HR = 1.962, 95% CI 1.630-2.362) with mild heterogeneity (I^2^ = 34.9%) and short DFS (HR = 2.050, 95% CI 1.293-3.249). High TP73-AS1 expression only in bladder cancer indicated long OS (HR = 0.400, 95% CI 0.180-0.880).

Subgroup analysis of the association between TP73-AS1 and OS was performed by examining TP73-AS1 expression in cancer tissue (high or low), cancer type (digestive system or non-digestive system), analysis method (multivariate analysis or univariate analysis) and NOS score (≥7 or <7), as shown in Table 2. The result of each subgroup was not significantly changed. Subgroup analysis revealed that differential expression of TP73-AS1 in cancer tissues was the potential source of heterogeneity.

**Table 2:** Subgroup analysis of the pooled HRs for OS.

Subgroup analysis	Number of studies	Number of patients	HR (95% CI)	P value	Heterogeneity	
					I^2^	P value
Overall	15	1047	1.962 (1.630-2.362)	p <0.001	34.9%	0.09
**Expression in cancer tissue**						
High	14	919	2.151 (1.777-2.603)	p <0.001	0.0%	0.971
Low	1	128	0.400 (0.181-0.884)	0.024	NA	NA
Cancer type						
Digestive system	6	431	2.124 (1.629-2.770)	p <0.001	0.0%	0.671
Non-digesstive system	9	616	1.837 (1.230-2.744)	0.003	54.6%	0.024
**Analysis method**						
Multivariate analysis	6	415	2.452 (1.816-3.310)	p <0.001	0.0%	0.653
Univariate analysis	9	632	1.709 (1.350-2.164)	p <0.001	45.8%	0.064
NOS score						
≥7	11	791	1.803 (1.449-2.244)	p <0.001	39.2%	0.088
<7	4	256	2.437 (1.716-3.461)	p <0.001	0.5%	0.389

**Figure 3:**
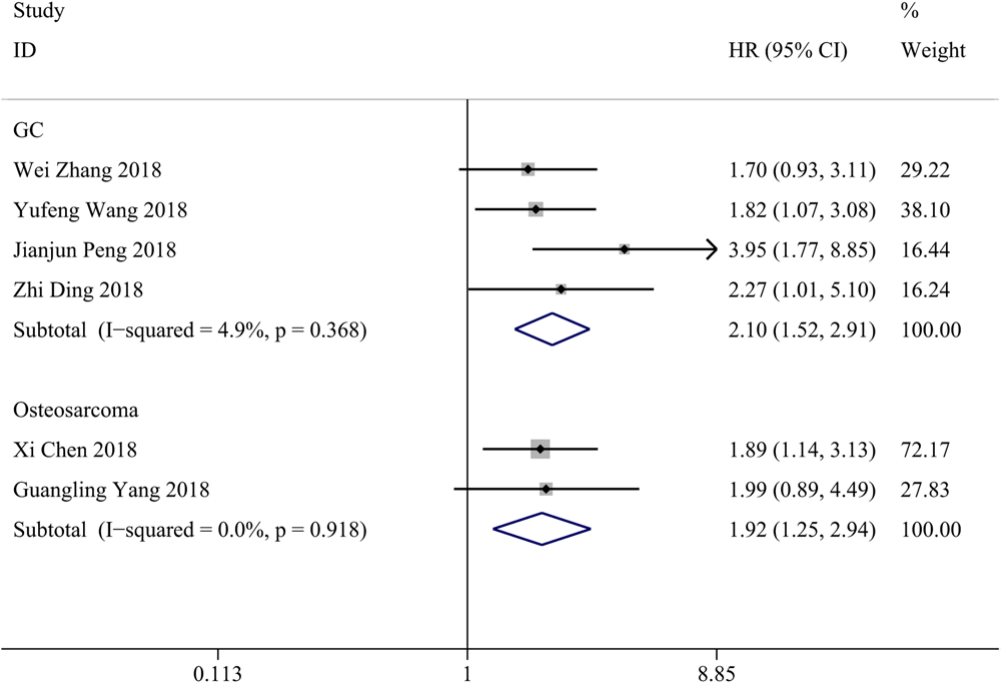
Forest plots for association of TP73-AS1 expression with overall survival (OS) in gastric cancer (GC) and osteosarcoma.

Four studies focusing on GC showed that high TP73-AS1 expression markedly predicted poor OS (HR = 2.101, 95% CI 1.516-2.911). Two studies on osteosarcoma demonstrated that high TP73-AS1 expression was significantly correlated with short OS (HR = 1.920, 95% CI 1.253-2.942). More details are provided in Fig. 3.

### Association between TP73-AS1 and clinicopathological features

**Figure 4:**
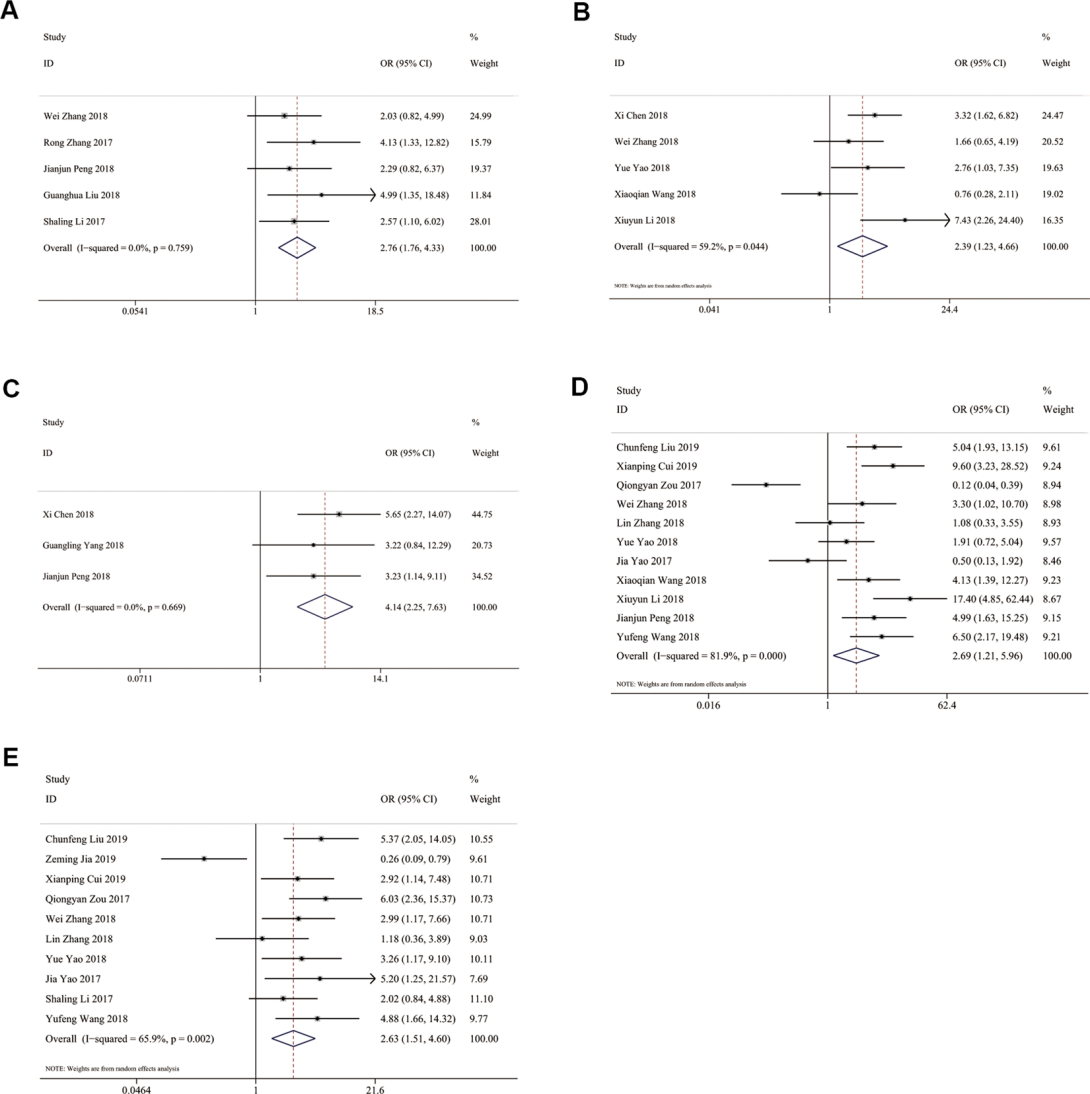
Forest plots for association of TP73-AS1 expression with clinicopathological features: (**A**) Tumor size; (**B**) Histological grade; (**C**) Distant metastasis; (**D**) Lymph node metastasis; (**E**) TNM stage.

The results demonstrated that high TP73-AS1 expression was not significantly associated with the age or gender of cancer patients (age: OR = 1.082, 95% CI 0.817-1.433; gender: OR = 1.034, 95% CI 0.772-1.385). As shown in Fig. 4, high TP73-AS1 expression was significantly related to large tumor size (OR = 2.759, 95% CI 1.759-4.330), advanced histological grade (OR = 2.394, 95% CI 1.231-4.656), lymph node metastasis (OR = 2.687, 95% CI 1.211-5.962), distant metastasis (OR = 4.145, 95% CI 2.252-7.629) and advanced TNM stage (OR = 2.633, 95% CI 1.507-4.601).

**Figure 5:**
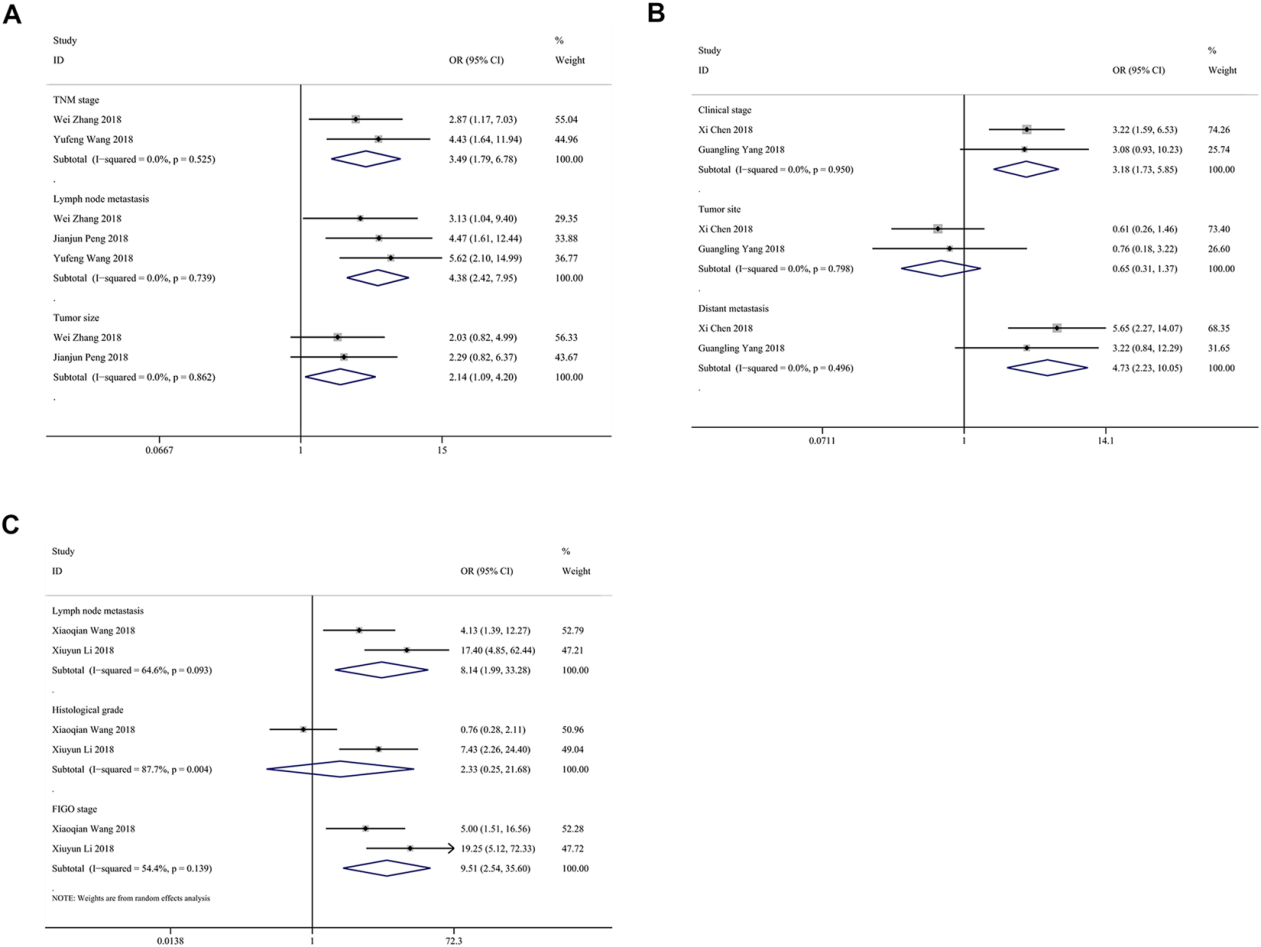
Forest plots for association of TP73-AS1 expression with clinicopathological features in 3 cancers: (**A**) Gastric cancer; (**B**) Osteosarcoma; (**C**) Ovarian cancer.

In Fig. 5, studies on GC showed that high TP73-AS1 expression was significantly associated with lymph node metastasis (OR = 3.792, 95% CI 1.793-8.018) and large tumor size (OR = 2.137, 95% CI 1.087-4.203) in GC patients. Two articles on breast cancer demonstrated that high TP73-AS1 expression was significantly linked with large tumor size (OR = 4.201, 95% CI 1.961-8.999), advanced TNM stage (OR = 5.764, 95% CI 2.637-12.598) and the absence of lymph node metastasis (OR = 0.234, 95% CI 0.057-0.966) but not with ER status (OR = 1.220, 95% CI 0.596-2.497) or PR status (OR = 1.700, 95% CI 0.828-3.491). Two studies on osteosarcoma supported that high TP73-AS1 expression was notably associated with advanced clinical stage (OR = 3.184, 95% CI 1.732-5.854) and distant metastasis (OR = 4.730, 95% CI 2.225-10.053) but not with tumor site (OR = 0.650, 95% CI 0.309-1.366). Two studies concerning ovarian cancer revealed an association of TP73-AS1 with lymph node metastasis (OR = 6.616, 95% CI 3.241-13.504) and histological grade (OR = 2.129, 95% CI 1.046-4.336). Moreover, two articles showed a significant association of TP73-AS1 with lymph node metastasis (OR = 8.139, 95% CI 1.990-33.282) and advanced FIGO stage (OR = 9.514, 95% CI 2.542-35.604) but not with histological grade (OR = 2.333, 95% CI 0.251-21.682).

### Publication bias and sensitivity analysis

**Figure 6:**
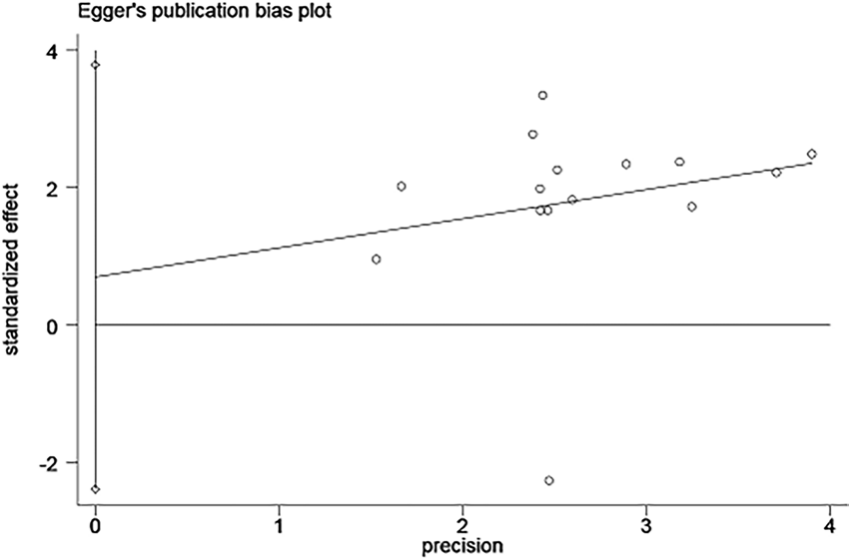
Egger’s test for publication bias of results of overall survival (OS).

Egger’s test was conducted to explore a possible publication bias. As shown in Fig. 6, the symmetrical Egger’s plot of all eligible studies for OS showed no significant publication bias(p = 0.633).

**Figure 7:**
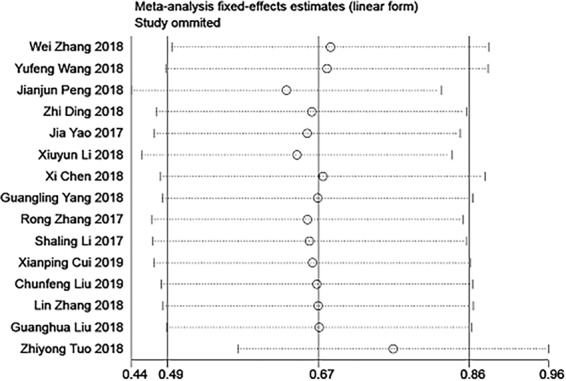
Sensitivity analysis for studies about OS by omitting each study sequentially.

Sensitivity analysis was performed to estimate the stability of the original OS results. The robustness of the meta-analysis conclusions was confirmed because the results were not altered significantly when any study was eliminated (Fig. 7).

## Discussion

A large number of articles have identified that lncRNAs such as lncRNA PVT1, lncRNA HULC, and lncRNA CRNDE are closely related to the initiation and progression of various cancers and could serve as prognostic biomarkers^28–30^. TP73-AS1 was initially described in a study on oligodendroglial tumors, which revealed that TP73-AS1 was downregulated in tumor samples mainly via epigenetic modifications and chromosome 1p loss, and the knockdown of TP73-AS1 induced cisplatin resistance in glioma cells^31^. High TP73-AS1 expression was associated with larger tumor size, advanced WHO stage and a shorter OS in glioma patients^23^. Tuo et al. reported that low TP73-AS1 expression predicted unfavorable survival and diminished bladder cancer cell proliferation, migration and invasion but facilitated apoptosis^10^. In contrast, TP73-AS1 promoted oesophageal cancer cell proliferation and inhibited apoptosis^32^. Knockdown of TP73-AS1 inhibited the vasculogenic mimicry formation of triple-negative breast cancer cells by targeting the miR-490-3p/TWIST1 axis^33^. Liu et al. revealed that TP73-AS1, upregulated ccRCC tissues, predicted the prognosis of ccRCC patients and promoted ccRCC cell proliferation and invasion but inhibited apoptosis^26^. These discrepancies show the different roles of TP73-AS1 in multiple cancers.

In this meta-analysis, nineteen eligible studies with 1329 cancer patients were included. Pooled results indicated that TP73-AS1 overexpression was significantly associated with poor OS and DFS. The association of TP73-AS1 with OS was robust in either gastric cancer or osteosarcoma. Subgroup analysis of OS indicated no significant change in any subgroup. Furthermore, high TP73-AS1 expression was markedly related to larger tumor size, lymph node metastasis, distant metastasis, advanced TNM stage and histological grade but not with age or gender.

Furthermore, we payed attention to potential targets and pathways of TP73-AS1 in the included cancers (Table 3). TP73-AS1 functioned as an oncogenic lncRNA in most cancers except bladder cancer and CRC. In this meta-analysis, high TP73-AS1 expression was associated with long OS only in bladder cancer. Tuo et al. found that in bladder cancer overexpression of TP73-AS1 could inhibit cell growth, arrest cell cycle, reduce cell migration and invasion, promote cell apoptosis, and diminish EMT by inhibiting the expression of vimentin, snail, MMP-2, and MMP-9 and upregulating the expression of E-cadherin^10^. On the contrary, TP73-AS1 could promote tumor progression through regulating HMGB1/RAGE pathway in GC, brain glioma and HCC. TP73-AS1 could target miR-142 both in osteosarcoma and brain glioma. In addition, TP73-AS1 was linked to chemoresistance in both GC and glioblastoma. TP73-AS1 might not only act as a biomarker of chemosensitivity, but also as a novel therapeutic target.

**Table 3:** Summary of potential targets and pathways of TP73-AS1.

Cancer type	Expression	Potential target	Pathway	References
GC	Upregulated	NA	Cell proliferation, apoptosis, invasion and metastatic properties; tumorigenesis; cisplatin resistance; Bcl-2/caspase-3 pathway; EMT; WNT/β-catenin signaling pathway; HMGB1/RAGE signaling pathway; miR-194-5p/SDAD1 axis	^9,16–18^
Breast cancer	Upregulated	miR-200a; miR-490-3p	Cell invasion, migration and vasculogenic mimicry; TFAM; TP73-AS1/miR-200a/ZEB1 regulating loop; miR-490-3p/TWIST1 axis	^13,19,33^
Ovarian cancer	Upregulated	p21; EZH2	Cell proliferation, apoptosis, invasion and migration; tumor growth; MMP2 and MMP9	^20,21,36^
Osteosarcoma	Upregulated	miR-142	Cell proliferation, migration and invasion; tumor growth; TP73-AS1/miR-142/Rac1 signaling pathway	^12,22^
Brain glioma	Upregulated	miR-142; miR-124	Cell proliferation and invasion; temozolomide resistance; HMGB1/RAGE pathway; ALDH1A1; TP73-AS1/miR-124/iASPP axis	^23,37,38^
HCC	Upregulated	miR-200a	Cell proliferation; HMGB1/RAGE pathway and NF-кB expression	^11^
Pancreatic cancer	Upregulated	miR-141	Cell migration, invasion and metastasis; BDH2	^24^
LAD	Upregulated	NA	Cell proliferation, apoptosis, migration and invasion; tumor growth and metastasis; PI3K/AKT pathway	^25^
NSCLC	Upregulated	miR-449a	Cell growth and cycle progression; TP73-AS1/miR-449a/EZH2 axis	^19^
Bladder cancer	Downregulated	NA	Cell proliferation, apoptosis, migration and invasion; EMT	^10^
ccRCC	Upregulated	KISS1	Cell proliferation, invasion and apoptosis; PI3K/Akt/mTOR pathway	^26^
CCA	Upregulated	NA	Cell proliferation, apoptosis and metastatic properties; tumor growth	^13^
CRC	Downregulated	miR-103; miR-194	Cell proliferation, apoptosis migration and invasion; tumor growth; TP73-AS1/miR-103 axis; PTEN; TP73-AS1/miR-194/TGFα axis	^27,39^

The limitations of this meta-analysis deserve to be mentioned. First, each study was conducted in China, which means that the results need to be verified by studies from other countries. Second, most studies had a small sample size (n < 100), which might result in inflated estimates of effect size. Third, the cut-off values were not available in 5 studies. Finally, we calculated the HRs from Kaplan-Meier curves in 12 studies, which may have affected the accuracy of the results.

## Conclusion

High TP73-AS1 expression in multiple cancers predicted poor OS, poor DFS, larger tumor size, lymph node metastasis, distant metastasis, advanced TNM stage and histological grade. TP73-AS1 might be a novel prognostic biomarker and therapeutic target for cancers. More high-quality studies with a large sample size and a standardized methodologic design are required to further certify the prognostic value of TP73-AS1 in cancers.

## Data Availability

All data are included in the article.
